# Efficacy of universal bonding agents as desensitizers: in vitro and in vivo studies

**DOI:** 10.1186/s12903-026-07767-z

**Published:** 2026-01-29

**Authors:** Xue Cai, Rongyu Cao, Xiaoyan Wang

**Affiliations:** 1 Department of Cariology and Endodontology, Peking University School and Hospital of Stomatology & National Center of Stomatology & National Clinical Research Center for Oral Diseases & National Engineering Research Center of Oral Biomaterials and Digital Medical Devices, Beijing, China; 2 Department of Operative Dentistry and Endodontics, The Affiliated Yantai Stomatological Hospital, Binzhou Medical University, Yantai, Shandong China

**Keywords:** Single bond universal, Hybrid coat, OptiBond universal, Hypersensitivity, Durability, Clinical trial

## Abstract

**Background:**

Dentin hypersensitivity (DH) is a highly challenging and persistent dental complaint. This study aimed to evaluate the in vitro durability of the universal dentin bonding agents Single Bond Universal (SBU) and OptiBond Universal compared with a specialized desensitizer, Hybrid Coat (HC), and to assess the desensitizing efficacy of SBU and HC in vivo over a 1-year follow-up period.

**Methods:**

Dental root samples were prepared and treated with the three agents. After 24 h, 3 months, and 6 months, longitudinal sections of the root samples were examined under scanning electron microscopy, and adhesive layer thicknesses were measured. A split-mouth, blinded, randomized controlled trial was then conducted to compare the efficacy of SBU and HC for DH relief, using the visual analog scale (VAS). Assessments were performed at baseline, immediately post-application, and at 1, 3, 6, 9, and 12 months of follow-up, using standardized cold air stimulus protocols. The difference in VAS scores between pre- and post-application was used to measure efficacy.

**Results:**

The adhesive layer created by SBU was thinner than that created by HC; however, the HC layer degraded more rapidly. After 6 months of water aging, root surfaces in the HC group were exposed, whereas those in the SBU group remained covered. The in vivo study demonstrated that SBU provided desensitizing efficacy comparable to that of HC over the 12-month period.

**Conclusions:**

Within the limitations of the present study, it can be concluded that the universal bonding agent SBU is effective and durable when applied as a desensitizer. Its efficacy is comparable to that of a dedicated desensitizing treatment.

**Trial registration:**

NCT07197437 ( https://www.clinicaltrials.gov), Date of Registration: 15/09/2025. (Retrospectively registered).

## Background

Dentin hypersensitivity (DH) is a common oral pain condition characterized by intense, transient pain resulting from stimulation of exposed dentin, typically in response to chemical, thermal, tactile, or osmotic stimuli. DH often diminishes patient quality of life because the pain is associated with frequent and tangible discomfort [1]. It occurs in both sexes, with higher prevalence among adults, and most commonly affects the facial surface of teeth in the cervical region [2]. The underlying cause of DH is exposure of dentinal tubules—microscopic channels that connect pulp to the tooth surface [3]. 

Management of DH requires a multifaceted approach, ranging from minimally invasive desensitizing toothpastes and agents to the ongoing introduction of novel biomaterials. Nevertheless, the most widely used clinical method remains the application of desensitizing agents, which act by occluding open dentinal tubules [3]. Evaluations of agent effectiveness are crucial for improving the quality of DH treatment.

Universal bonding agents, such as Single Bond Universal (SBU) and OptiBond Universal (OBU), have gained attention for their ability to reduce hypersensitivity by sealing dentinal tubules and reducing nerve activation [4] Although a few reports have demonstrated the desensitizing effects of universal bonding agents, most studies have evaluated only immediate or short-term outcomes [3, 5]. 

The present study compared the performances of universal bonding agents with that of Hybrid Coat (HC), a desensitizer widely used in clinical practice. It specifically examines the desensitizing efficacy and durability of universal bonding agents over a 6- to 12-month period, with the goal of determining whether these agents are as effective as HC for the long-term management of DH. The null hypothesis is that the desensitizing efficacy and durability of universal bonding agents are the same as that of Hybrid Coat.

## Methods

Deionized water was produced using a Milli-Q system (MilliporeSigma, Burlington, MA, USA). Unless otherwise specified, all solvents and reagents were obtained from Sigma-Aldrich (Sigma-Aldrich, St. Louis, MO, USA).

### In vitro aging of adhesive layers

Twenty-seven extracted sound human premolars were collected and stored in 0.9% NaCl at 4 °C until use. Soft tissues were removed prior to experimentation. Crowns were sectioned at the cementoenamel junction with a low-speed diamond saw (Isomet, Buehler, IL, USA). A 3-mm × 3-mm window was preserved 0.5 mm below the cementoenamel junction; the remaining root surface was coated with three layers of oil-based nail varnish. Specimens were randomly allocated to three groups based on the adhesive applied to the window area: HC (Sun Medical Dental Materials, Shiga, Japan), SBU (3 M, St. Paul, MN, USA), and OBU (Kerr Dental, Brea, CA, USA). Each group was further divided into three subgroups and immersed in 0.5% (w/v) chloramine-T aqueous solution at 37℃ for 24 h, 3 months, or 6 months, respectively (*N* = 3). After storage, specimens were longitudinally cleaved into equal halves. Each half was examined using a scanning electron microscope (SEM, SU8010, Hitachi, Tokyo, Japan). Adhesive layer thickness was measured by SEM at three standardized points: one at the center of the interface and two at 0.5 mm from the center (18 data points per subgroup).

### In vivo desensitization efficacy and durability of adhesives

A prospective, double-blind, randomized controlled trial with a split-mouth design was conducted among patients at the Department of Cariology and Endodontology, Peking University School and Hospital of Stomatology. Ethical approval was acquired from the Ethical Committee of Peking University School and Hospital of Stomatology (PKUSSIRB-202273039). Written informed consent was obtained from all participants. The study was non-invasive, ensured patient safety, and allowed voluntary withdrawal at any time. No adverse reactions were reported. The trial was conducted between March 2023 and March 2025.

#### Inclusion criteria

1. Participants aged ≥ 18 years who were able to provide written informed consent.
2. Participants confirmed as healthy according to the examiner, without clinically significant diseases that could interfere with study outcomes.
3. Participants with symptoms of dentin hypersensitivity affecting symmetrical teeth on both sides due to gingival recession.
4. Symptomatic teeth without caries, filling materials, or use as abutments for removable partial dentures.

#### Exclusion criteria

1. Individuals with dentin hypersensitivity caused by other factors (e.g., erosion or wedge-shaped defects).
2. Individuals with teeth exhibiting deep periodontal pockets (≥ 4 mm) or with a difference in gingival recession between the two symptomatic teeth of ≥ 1 mm.
3. Individuals who had received treatment or used toothpaste for dentin hypersensitivity within the previous 4 weeks.
4. Individuals who had undergone tooth bleaching or periodontal treatment within the previous 4 weeks.
5. Individuals who had undergone cervical restorative treatment.
6. Individuals who had taken nonsteroidal anti-inflammatory drugs or narcotic analgesics within the previous week.

#### Dentin sensitivity measurement methods

Tooth sensitivity was assessed using the visual analog scale (VAS), which classifies pain on a 0–10 scale. A score of 0 indicates no pain; <3 indicates mild, tolerable pain; 4–6 indicates pain that affects sleep but remains tolerable; and 7–10 indicates severe, intolerable pain.

Sensitivity was evaluated using a cold air test. Symptomatic teeth were isolated, and air was applied perpendicularly using an air-water syringe at a distance of 5 mm from the sensitive surface for 1 s. The VAS score was recorded. After 1 min, the test was repeated, and the VAS score was recorded again. If the two scores differed, the higher value was used.

#### Procedural details

After participant enrollment, tooth surfaces were cleaned; baseline VAS scores were recorded using the cold air test. One side of the symptomatic teeth was randomly assigned to treatment with HC, whereas the contralateral side was treated with SBU. Simple randomization was employed for group assignment, with the random sequence generated by a computer random number generator pre-operatively by the dental assistant. The operator retrieved only individual subject allocation results from the dental assistant in accordance with enrollment order, with no access to the full sequence—thus mitigating operator-mediated selection bias. After isolation, HC was applied with a coated cotton pellet and SBU was applied with a microbrush for 20 s; these applications were followed by gentle air-drying with an air-water syringe for 5 s and light curing for 20 s. Participants were able to be blinded. Due to distinct application protocols for HC and SBU, operator masking was not feasible. All cold air test assessments, including baseline and outcome assessments, were conducted by an independent assessor masked to treatment assignments, thus ensuring the integrity of the blinding procedure. The assessor had no access to group allocation details from dental assistants and was segregated in a separate room during the intervention to preclude observation of material administration.

Follow-up evaluations were conducted immediately after treatment and at 1, 3, 6, 9, and 12 months. Dentin sensitivity was reassessed at each visit and recorded in terms of VAS score by the same blinded assessor.

Primary outcome was treatment efficacy (d). It was defined as the change in VAS score before and after desensitization. Outcomes were classified as ineffective when d < 2 and effective when d ≥ 2. The efficacy rate (ER) was calculated as: ER = number of effective teeth / total number of symptomatic teeth × 100%.

When outcomes were identified as ineffective (d < 2), the participants would receive other treatment procedure to solve the dentin hypersensitivity, according to ethical requirements. The trial would automatically terminate.

### Statistical analysis

Sample size calculation was performed using the formula for equivalence test sample size estimation. Based on the findings of the previous studies [3, 5] and our pilot study, the difference in effective rates between the two groups (θ) was set to zero. The following formula is commonly employed for equivalence test sample size estimation:[6]

$$N=2\left[\left(Z_{1-\alpha} + Z_{1-\beta/2}\right)\left(\sigma/\triangle-\theta\right)\right]^{2}$$

The treatment efficacy (d) was the primary outcome. The equivalence margin (Δ) is assumed to be 2. The standard deviation (σ) of the data was set to be 2. With an 80% power and a 90% confidence interval, the required sample size was estimated to be 18 in total. Considering a dropout rate of 10% – 20%, the total sample size needs to be increased to 20–22 (corresponding to 10–11 teeth per group).

Data were processed and analyzed using SPSS software (Version 27). Categorical variables, such as sex, were expressed as percentages. VAS scores normality was assessed using the Kolmogorov–Smirnov test. Differences in VAS scores among and within adhesive groups across time intervals were evaluated by Repeated Measures ANOVA. A *p*-value < 0.05 was considered statistically significant.

## Results

### In vitro aging of adhesive layers

**Fig. 1:**
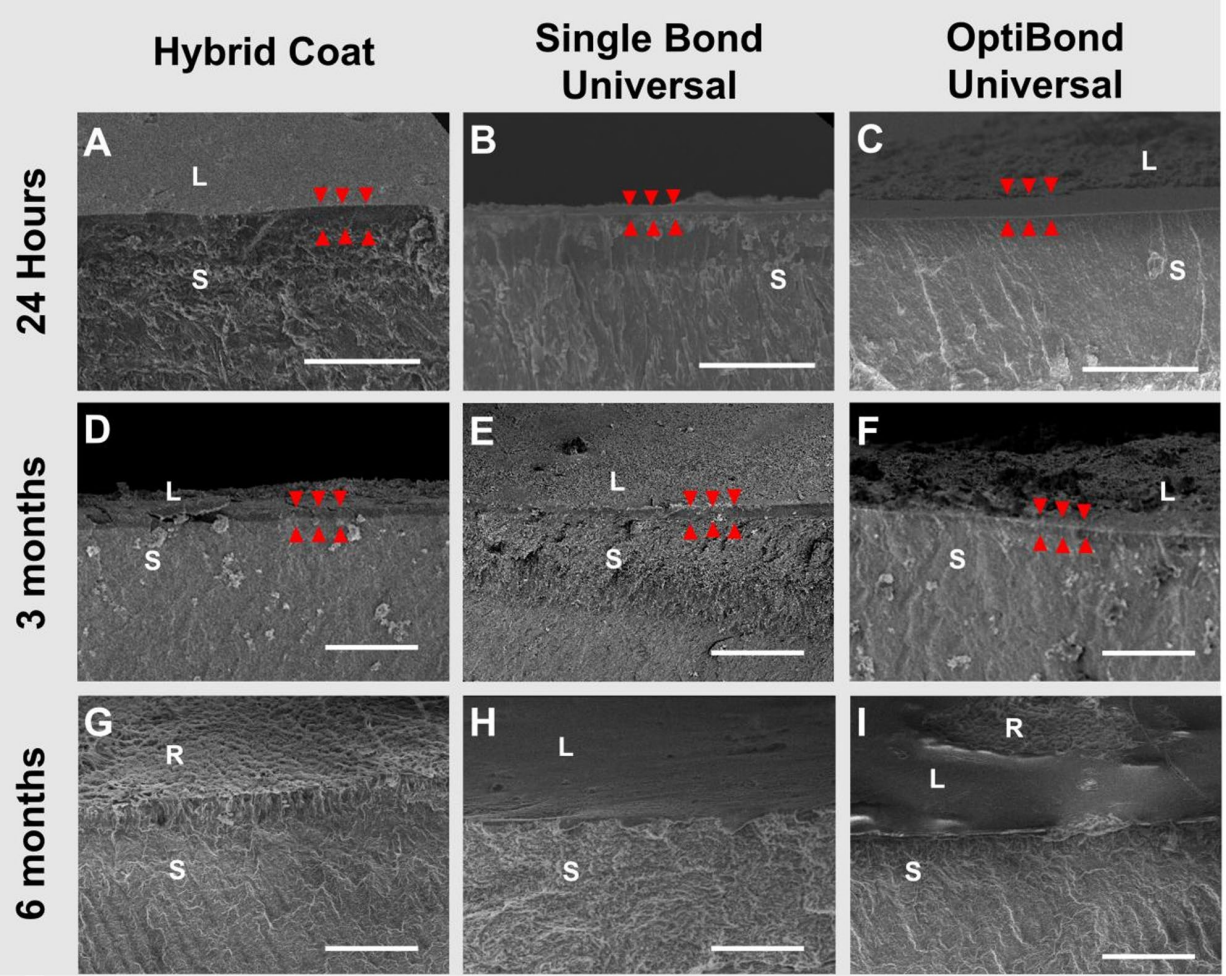
Representative longitudinal sections under SEM of samples after application of different adhesives and water aging. **A** Hybrid Coat, 24 h; **B** Single Bond Universal, 24 h; **C** OptiBond Universal, 24 h; **D** Hybrid Coat, 3 months; **E** Single Bond Universal, 3 months; **F** OptiBond Universal, 3 months; **G** Hybrid Coat, 6 months; **H** Single Bond Universal, 6 months; **I** OptiBond Universal, 6 months. Scale bar = 50 μm. L: adhesive layer; S: longitudinal section of the root; R: root surface; Arrowheads: adhesive layer thickness

Representative SEM images obtained after 24 h, 3 months, and 6 months of adhesive application are shown in Fig. 1. Red arrowheads indicate the adhesive layer thickness. At 24 h, the adhesive layers produced by HC (5.77 ± 1.12 μm) and OptiBond Universal (4.32 ± 0.56 μm) were significantly thicker than that of SBU (2.81 ± 0.08 μm) (*p* < 0.05). After 3 months of water aging, the HC layer decreased to 2.02 ± 0.98 μm and no longer adhered tightly to the root surface, whereas SBU and OptiBond Universal measured 1.80 ± 0.06 μm and 1.43 ± 0.11 μm, respectively (*p* > 0.05). After 6 months of water aging, the HC layer had completely disappeared, leaving the root surface exposed. The SBU layer was reduced to approximately 0.2 μm. Although this was too thin for accurate measurement, the root surfaces remained fully covered. OptiBond Universal layers were thinner than those of SBU, as large areas of the root surface were exposed in the OptiBond Universal group.

### In vivo desensitization efficacy and durability of adhesives

Fourteen participants were enrolled in the clinical study, and none were excluded. All participants completed the 12-month follow-up. In total, 24 teeth each were assigned to the HC and SBU groups. The power analysis based on the observed effect size and the actual sample size was performed. The actual power was approximately 99%, which was higher than the preset 80%. Patient demographics are summarized in Table 1. Baseline demographic features, including sex distribution, mean age, tooth position, and mean VAS scores, were comparable between groups (*p* > 0.05). No adverse events were reported during the 12-month follow-up period.

**Table 1:** Baseline participant characteristics

Participants	Variables		Overall characteristics
	Age(years)	Mean(SD)	40.14
		Median	39.00
		Min-Max	27-60
	Sex	Male	6
		Female	8
Type of DH teeth	Variables	Group HC	Group SBU
	Premolars	14	14
	Molars	10	10
Mean VAS score at screening (SD)		5.17(1.64)	5.00(1.54)

*DH* dentin hypersensitivity, *SD *standard deviation

**Fig. 2:**
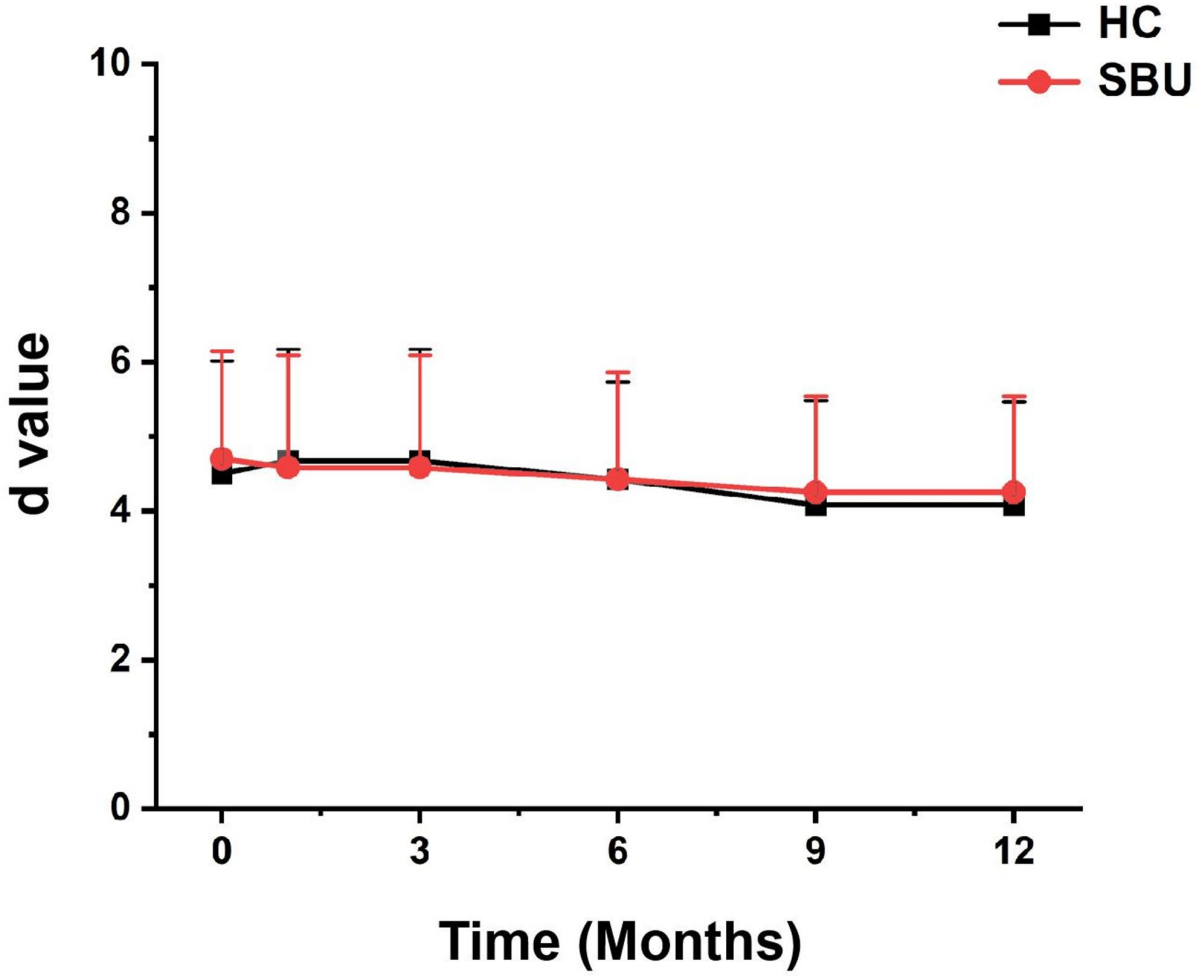
Treatment efficacy (d value) of SBU and HC over a 1-year follow-up period

Effectiveness rates in both groups were 100% immediately after treatment. As shown in Fig. 2, the mean d value in the HC group was 4.50 ± 1.47, whereas it was 4.71 ± 1.40 in the SBU group (*p* > 0.05). Over the 12-month follow-up period, d values gradually decreased to 4.17 ± 1.34 in the HC group and 4.21 ± 1.25 in the SBU group.

There was no statistically significant difference between the two groups (F = 0.12, *p* > 0.05), with a negligible effect size (η_p_^2^ = 0.003). In contrast, the main effect of time was statistically significant (F = 3.85, *p* < 0.05), and the large effect size (η_p_^2^ = 0.082), indicated that the outcome indices of both groups decreased significantly over time, which had practical clinical implications. Furthermore, there was no significant interaction between group and time (F = 0.09, *p* > 0.05, η_p_^2^ = 0.002), suggesting that the decreasing trends of the two groups were consistent throughout the observation period. Despite this reduction, effectiveness rates remained 100% in both groups after 12 months.

To verify the robustness of the findings, a robust repeated-measures ANOVA with Huber’s M-estimator was performed. Consistent with the traditional analysis, no statistically significant group difference (F = 0.11, *p* > 0.05, η_p(r)_^2^ = 0.002) or group-time interaction (F = 0.08, *p* > 0.05, η_p(r)_^2^ = 0.001) was observed. Meanwhile, the main effect of time remained statistically significant (F = 3.79, *p* < 0.05, η_p(r)_^2^ = 0.079), confirming that the outcome indices of both groups decreased significantly over time. These results indicated that our conclusions were not affected by potential outliers or the normality of the data.

## Discussion

The present study evaluated the clinical efficacy of universal dentin bonding agents as less technique-sensitive desensitizing agents. During the management of DH, application simplicity and efficiency are critical, particularly in clinical settings where time and resources are limited. By providing empirical evidence in support of universal adhesives, this study demonstrates their value as effective and practical alternatives for DH treatment [3]. 

SBU and OBU are seventh-generation adhesives that can be applied in either the etch-and-rinse or the self-etch mode. The self-etch mode requires only direct application of the adhesive to the tooth surface, eliminating the need for prior etching. This approach simplifies the bonding process and reduces technique sensitivity [7]. Therefore, the clinical applications of universal adhesives have considerably expanded. HC is also essentially a one-step self-etch adhesive [8]. All of these materials form hybrid layers over exposed dentin surfaces, thereby blocking communication between dentinal tubules and the external environment. Consequently, adhesive layer retention corresponds to the duration of dentinal tubule sealing, which in turn determines the durability of the desensitizing effect.

In the in vitro study, three teeth were allocated per group in this in vitro experiment to balance rationality and practicality. This sample size satisfied the minimum requirement for parametric tests, enabling verification of the effects of experimental treatments at minimal time and consumable costs. During the measurement, each dental root sample was divided into two halves. Although the measurement points may appear roughly symmetrical from a macroscopic perspective, they cannot completely coincide under SEM. Therefore, each tooth has six measurement points, and each group has 18 data points. This experiment revealed that whereas the adhesive layer formed immediately by HC was thicker, it rapidly degraded under water-aging conditions. In contrast, the adhesive layer formed by SBU, although initially thinner, remained intact after 6 months of water aging. OBU demonstrated an intermediate performance between HC and SBU. A previous study showed that the microtensile bond strength of SBU to dentin in self-etch mode reaches 47.44 ± 10.91 MPa at 24 h and maintains 46.34 ± 6.22 MPa after 6 months [9]. In comparison, the microtensile bond strength of HC is only approximately 6.7 ± 1.5 MPa [10]. Furthermore, one study showed that the HC layer could be worn away before 20,000 cycles of toothbrush abrasion [11]. These findings may explain why SBU layers persisted longer than HC on root surfaces.

Limited by the conditions of clinical research, such as the number of participants recruited and the ethical consideration of the treatment effectiveness, SBU was selected for in vivo evaluation, for its stronger anti-aging properties, which could be more promising; HC served as the control specialized desensitizer. A key feature of this research is its adoption of a blind methodology, a critical aspect in clinical trials aimed at reducing reporting bias. By ensuring that the assessor was not aware of the treatment allocation, the study significantly mitigates the potential for subjective influence on the outcomes, thereby enhancing the integrity and validity of the results. Another key feature is its adoption of a spit-mouth methodology. It removes a lot of inter-individual variability from the estimates of the treatment effect. The pain caused by dentin hypersensitivity is transmitted through the dental pulp nerve. The anatomy of the dental pulp nerve determines that its pain cannot be confused on both sides, but only on the upper and lower sides. Therefore, the contralateral neural sensitization that commonly appears in pain research was not required to be considered here.

The duration of desensitizing efficacy was similar between the two groups. These findings suggest that SBU can be used directly for dentin desensitization, providing outcomes comparable to those of specialized desensitizers. Furthermore, SBU forms a thinner film that does not interfere with subsequent procedures (e.g., crown cementation), and thus has broader indications than HC, making it an effective option for dentin hypersensitivity relief when acquisition of a specialized desensitizer is unnecessary.

However, the effect of SBU in vivo was not significantly superior to that of HC. One possible explanation is that the physical occlusion of dentinal tubules by the layers formed by adhesives is only one aspect of their mechanism of action. More importantly, MDP in SBU and 4-META in HC can both penetrate deeply into the dentinal tubules, bind to hydroxyapatite, and alter the permeability of dentinal tubules [12]. Therefore, the loss of superficial layers may not necessarily lead to treatment failure. Another possible explanation is the limited sample size, which is also the limitation of the present study. With the current sample size, the study may fail to detect subtle differences or rare adverse events. Since the occurrence of dentin hypersensitivity in bilaterally symmetric tooth sites is relatively rare, multicenter clinical trials may be required to expand the sample size.

## Conclusions

Within the limitations of the present study, it can be concluded that the universal bonding agent SBU is effective and durable when applied as a desensitizer. Its efficacy is comparable to that of a dedicated desensitizing treatment.

## Data Availability

The datasets used and/or analyzed during the current study are available from the corresponding author on reasonable request.
